# Multifunctional applications and research advances of low-molecular-weight heparin

**DOI:** 10.3389/fphar.2025.1585762

**Published:** 2025-05-21

**Authors:** Yanru Zhang, Shuixian Guo, Jingchao Xu

**Affiliations:** School of Medicine, Henan Polytechnic University, Jiaozuo, China

**Keywords:** low-molecular-weight heparin, anticoagulant effect, clinical application, mechanism of action, pharmacokinetics, personalized treatment

## Abstract

Low-molecular-weight heparin (LMWH) is a class of anticoagulant drugs derived from the controlled depolymerization of heparin. LMWH possesses a lower molecular weight and a shorter glycan chain length than unfractionated heparin (UFH), resulting in higher bioavailability, a more predictable pharmacokinetic profile, and consequently, a more convenient administration route. These characteristics make LMWH a primary choice for thromboprophylaxis of deep vein thrombosis. LMWH is clinically indicated for the prevention and treatment of venous thromboembolic diseases. Its role in obstetric complications, inflammation modulation, and antitumor therapy is also increasingly being recognized. The objective of this review was to systematically summarize the progress of LMWH research and to thoroughly explore its mechanisms of action and clinical indications. By analyzing the advantages and disadvantages of LMWH, evaluating its safety and adverse reactions, discussing the challenges in its clinical application, and proposing future research directions, in this review, we aim to promote the application and development of LMWH in a broader range of fields.

## 1 Introduction

LMWH represents a class of anticoagulant agents produced through the chemical or enzymatic depolymerization of heparin. These agents possess a molecular weight ranging 4,000–8,000 Da and a glycan chain length of 6–12 disaccharide units (Li et al., 2014). The structural heterogeneity of LMWH is characterized by variations in the amounts of sulfate and acetamido–sulfate groups, as well as a distinctive internal ether structure (Chandarajoti et al., 2016). LMWH demonstrates higher bioavailability (Fareed et al., 2003), a more predictable pharmacokinetic profile (Nowak-Göttl et al., 2008), and more simplified subcutaneous administration (Robertson and Jones, 2017) than conventional unfractionated heparin (UFH). Consequently, it has rapidly emerged as one of the primary choices for clinical anticoagulation therapy since its development in 1976. Currently, LMWH is primarily used in the prevention and treatment of venous thromboembolism (VTE), including deep vein thrombosis and pulmonary embolism (PE) (Prandoni, 2008). Additionally, with advancements in basic research, the roles of LMWH in regulating inflammation (Hochart et al., 2006), antineoplastic therapy (Du, 2019), and addressing pregnancy-related complications (Cruz-Lemini, 2022) have garnered increasing attention.

This review systematically consolidates the current knowledge on LMWH. We critically evaluate the molecular mechanisms mediating its anti-inflammatory, antitumor, and antiviral effects while also addressing its applications in specialized populations, namely, pregnant women, cancer patients, and individuals with renal impairment. By synthesizing preclinical insights and clinical trial data, in this article, we aim to elucidate the dual therapeutic landscape of LMWH, encompassing both the anticoagulant and pleiotropic effects, assess the safety challenges and pharmacokinetic variability that complicates outcomes in diverse patient cohorts, and identify unmet needs in LMWH research, such as personalized dosing strategies and novel drug delivery systems, to optimize its therapeutic efficacy.

Through this comprehensive analysis, we aim to foster interdisciplinary collaborations and systematically direct research efforts into LMWH’s untapped potential, thereby ultimately facilitating its seamless incorporation into precision medicine paradigms.

## 2 Mechanism of action and pharmacokinetic characteristics

### 2.1 Anticoagulation

**FIGURE 1:**
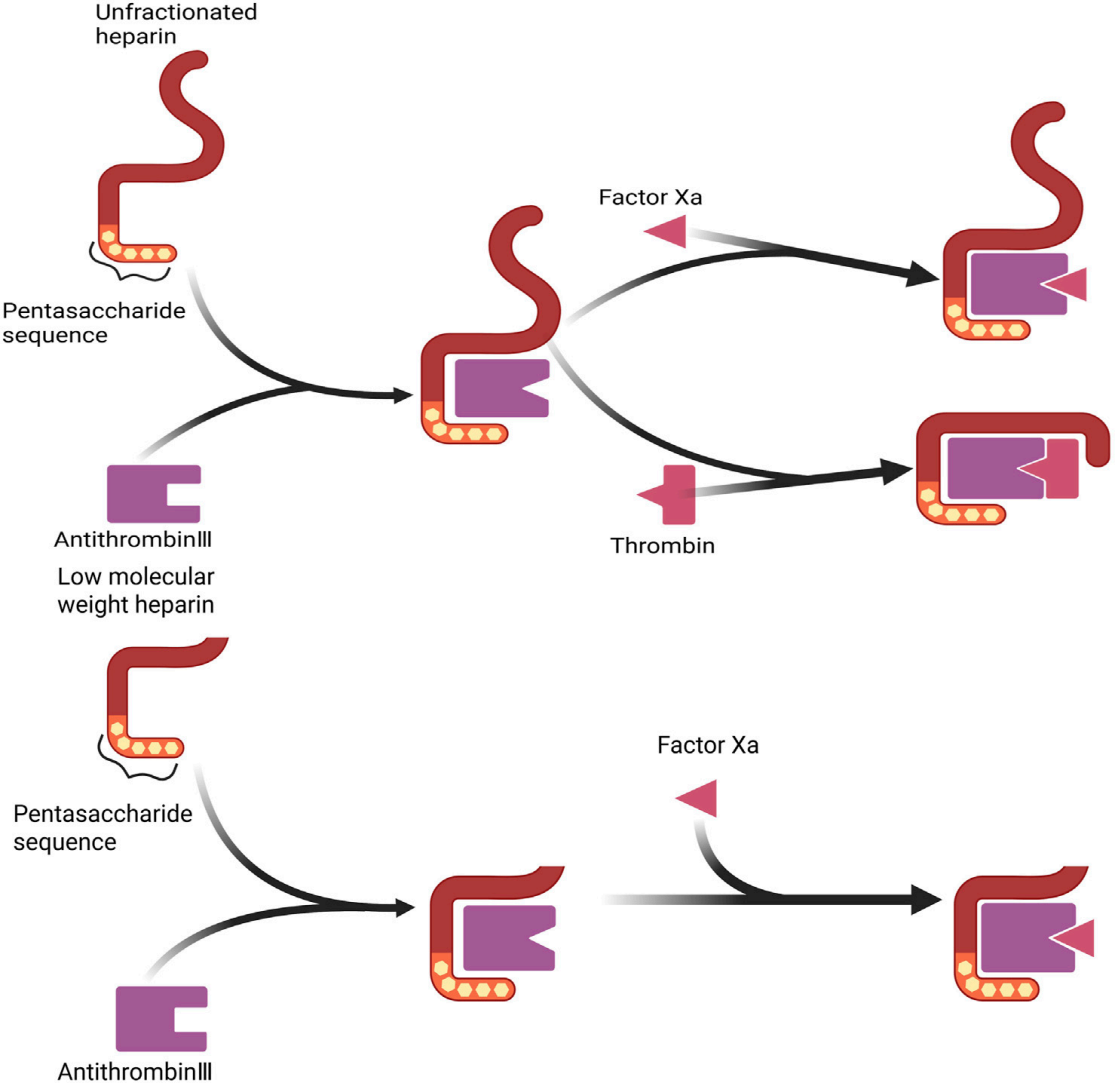
Comparison of UFH and LMWH anticoagulation mechanisms. This figure demonstrates the difference in anticoagulation mechanisms between UFH and LMWH. Normal heparin is able to inhibit both thrombin (factor IIa) and factor Xa, whereas low-molecular-weight heparin mainly inhibits factor Xa and has a weaker inhibitory effect on thrombin. This selective inhibition gives LMWH a lower risk of bleeding.

LMWH is a pivotal agent in anticoagulation therapy, and it is characterized by its relatively low anticoagulant activity and significant antithrombotic effects. It is extensively utilized in the prevention and treatment of various thrombotic disorders. LMWH primarily interacts with antithrombin III (ATIII) *via* its distinctive pentasaccharide sequence, thereby potentiating the inhibitory actions of ATIII on Factor Ⅱa and Factor Xa. This interaction effectively reduces blood coagulation and mitigates the risk of thrombosis (Sharma, 1998). However, due to the lower molecular weight, most LMWH molecules contain fewer than 18 monosaccharide units. Consequently, LMWH cannot inhibit thrombin by forming a potent complex with both ATIII and thrombin (Figure 1), as UFH can (Weitz, 1997). This limitation results in a relatively low inhibitory effect on thrombin. The ratio of anti-factor Xa to anti-factor IIa activity of UFH is approximately 1:1, whereas that of LMWH typically ranges from 2:1 to 4:1 (Gerotziafas et al., 2007). The selective inhibition of coagulation factor Xa by LMWH confers antithrombotic activity with a relatively low risk of bleeding, as thrombin inhibition is a major contributor to bleeding syndromes (Rühl et al., 2012). This property has facilitated the broader clinical application of LMWH.

### 2.2 Anti-inflammatory

In addition to its well-established role in anticoagulation therapy, LMWH has gained considerable research interest in recent years for its anti-inflammatory properties.

**FIGURE 2:**
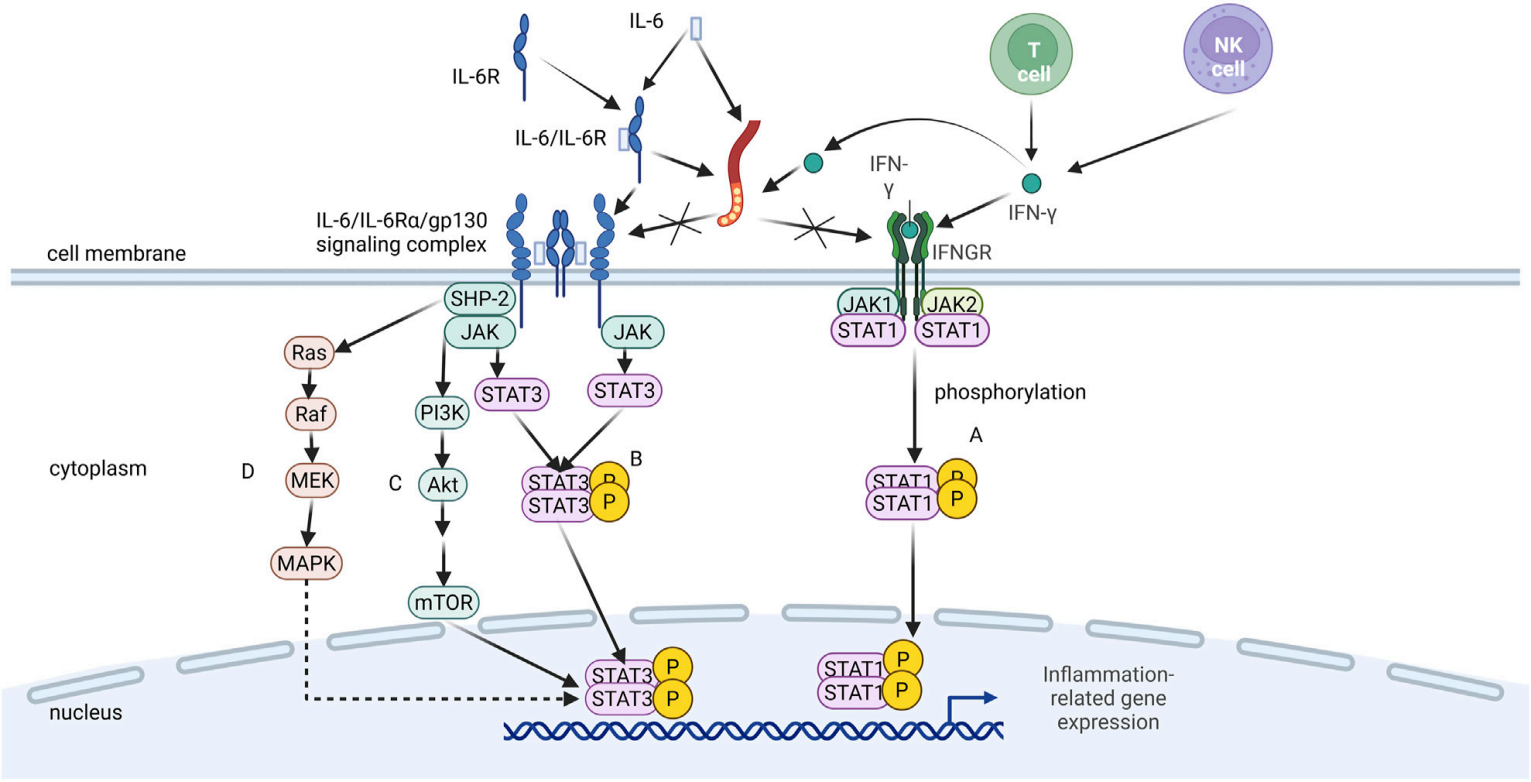
Mechanism of LMWH in blocking pro-inflammatory signaling pathways. LMWH binds to interleukin-6 (IL-6), interferon-y (IFN-y), and the IL-6/IL-6 receptor (IL-6R) complex, thereby competitively inhibiting their interaction with cell surface receptors (glycoprotein 130 gp130] and IFN-y receptor). This blockade prevents the activation of downstream intracellular signaling cascades, such as the JAK/STAT pathway, ultimately suppressing inflammatory responses.

IFN-γ is a cytokine produced by activated T cells and natural killer cells, which leads to the initiation and amplification of the inflammatory response by activating immune cells, promoting the secretion of inflammatory factors, and regulating gene expression (Figure 2A; Ivashkiv, 2018). LMWH binds IFN-γ with high affinity and completely inhibits interaction with its cellular receptor (Litov et al., 2021).

The IL-6/IL-6Rα/gp130 signaling complex is a cell-surface hexameric structure composed of cytokine IL-6, its specific receptor α-chain (IL-6Rα), and the signaling molecule gp130 (Skiniotis et al., 2005). This complex plays a pivotal role in inflammatory responses by activating multiple intracellular signaling pathways, including JAK–STAT (Figure 2B), PI3K–Akt (Figure 2C), and Ras–MAPK (Figure 2D). These pathways promote the production of inflammatory cytokines, activate immune cells, and regulate the acute-phase response and tissue repair, thereby driving inflammation and modulating the inflammatory process (Scheller, 2010). LMWH can modulate IL-6 bioactivity by binding to either IL-6 or the IL-6/IL-6Rα complex, thereby inhibiting the formation of the IL-6/IL-6Rα/gp130 signaling complex (Litov et al., 2021).

LMWH exerts its biological effects by interacting with the complement system, chemokines, and inflammatory molecules through its unique structural properties. Specifically, LMWH can bind to and inhibit multiple complement factors in both the classical and alternative pathways, such as the active C1 complex and C3-convertase. It also interferes with the assembly of the membrane attack complex, thereby reducing complement activation and terminal complement-mediated cytolysis, and protecting cells from complement-mediated damage (Vitiello and Ferrara, 2023).

In addition to its effects on the complement system, LMWH interacts with chemokines and prevents these molecules from binding to their specific receptors, thereby reducing signaling by inflammatory mediators. At the molecular level, LMWH reduces the activation of inflammatory molecules and regulates the expression and production of pro-inflammatory mediators including cytokines (e.g., IL-6), chemokines (e.g., MCP-1), and adhesion molecules (e.g., VCAM-1) by affecting the activity of the transcription factor NF-κB and inhibiting its translocation from the cytoplasm to the nucleus. These mechanisms collectively contribute to the anti-inflammatory effects of LMWH, which are independent of its anticoagulant activity (Vitiello and Ferrara, 2023).

The multiple anti-inflammatory mechanisms of action of LMWH have led to the demonstration of significant clinical potential in the treatment of inflammatory diseases (Shute, 2023; Podda, 2024), especially in situations where anticoagulation is required while a reduction in the inflammatory response is desired.

### 2.3 Protection of endothelial cells

Endothelial cells form a monolayer that lines the inner surfaces of the heart, blood vessels, and lymphatic vessels. These cells, which originate from the embryonic mesoderm, exhibit a diverse array of physiological functions. They are instrumental in angiogenesis, maintaining vascular integrity, modulating vascular tone, and participating in inflammatory responses and hemostasis (Dulak et al., 2013).

LMWH enhances endothelial-type nitric oxide synthase (eNOS) activity and increases nitric oxide (NO) production through the activation of the PI3K–Akt signaling pathway, an effect that is not dependent on calcium ions and provides an important protective mechanism for vascular endothelial cells, which contributes to the improvement of vascular endothelial function, reduction of platelet aggregation, and reduction of vascular inflammation, thus playing a positive role in vascular health (Feng et al., 2023).

Increased activity of heparinase, the only enzyme known to degrade heparan sulfate, damages the glycocalyx, leading to the loss of endothelial barrier function and vascular leakage. LMWH maintains the integrity of the endothelial cell glycocalyx by inhibiting heparinase activity and decreasing the production of heparin sulfate fragments, which protects the glycocalyx from degradation (Feng et al., 2023). In addition, LMWH can bind to heparan sulfate proteoglycans (HSPGs) on the surface of endothelial cells, and this binding action helps to stabilize the glycocalyx layer and resist glycocalyx detachment during inflammatory processes (Lipowsky and Lescanic, 2017).

LMWH exerts positive effects on endothelial cells through a variety of mechanisms, including activation of signaling pathways, inhibition of heparinase activity, and binding of HSPGs, all of which are essential for the maintenance of vascular health and prevention of related diseases.

### 2.4 Enhancement of mesenchymal stem cell function

Mesenchymal stem cells (MSCs) are a class of adult stem cells with multidirectional differentiation potential that are mainly derived from bone marrow, umbilical cord blood, and adipose tissue. They are capable of differentiating into various mesenchymal lineages such as bone, cartilage, muscle, and adipocytes (Yang, 2018). Adipose-derived mesenchymal stem cells (ASCs) play a role in anti-inflammatory and anti-fibrotic therapy by secreting a variety of anti-inflammatory and anti-fibrotic factors (Kotani, 2024). LMWH significantly enhances the function of MSCs, especially ASCs, including improving their migration capacity, increasing hepatocyte growth factor secretion, and upregulating the expression of factors associated with cell migration, thereby enhancing their anti-inflammatory and anti-fibrotic effects. LMWH-activated adipose-derived MSCs have shown promising therapeutic effects in mouse models of systemic lupus erythematosus (SLE) and bleomycin-induced systemic sclerosis (SSc) (Suzuka, 2022), making them potential therapeutic tools for the treatment of diseases such as SLE and SSc.

### 2.5 Potential mechanisms of action

LMWH has a variety of potential mechanisms of action due to its unique molecular structure and its ability to interconjugate with a wide range of biomolecules. This structure allows LMWH to not only play a role in anticoagulation therapy but may also exhibit a wide range of therapeutic effects in antiviral and antitumor applications.

#### 2.5.1 Antiviral

**FIGURE 3:**
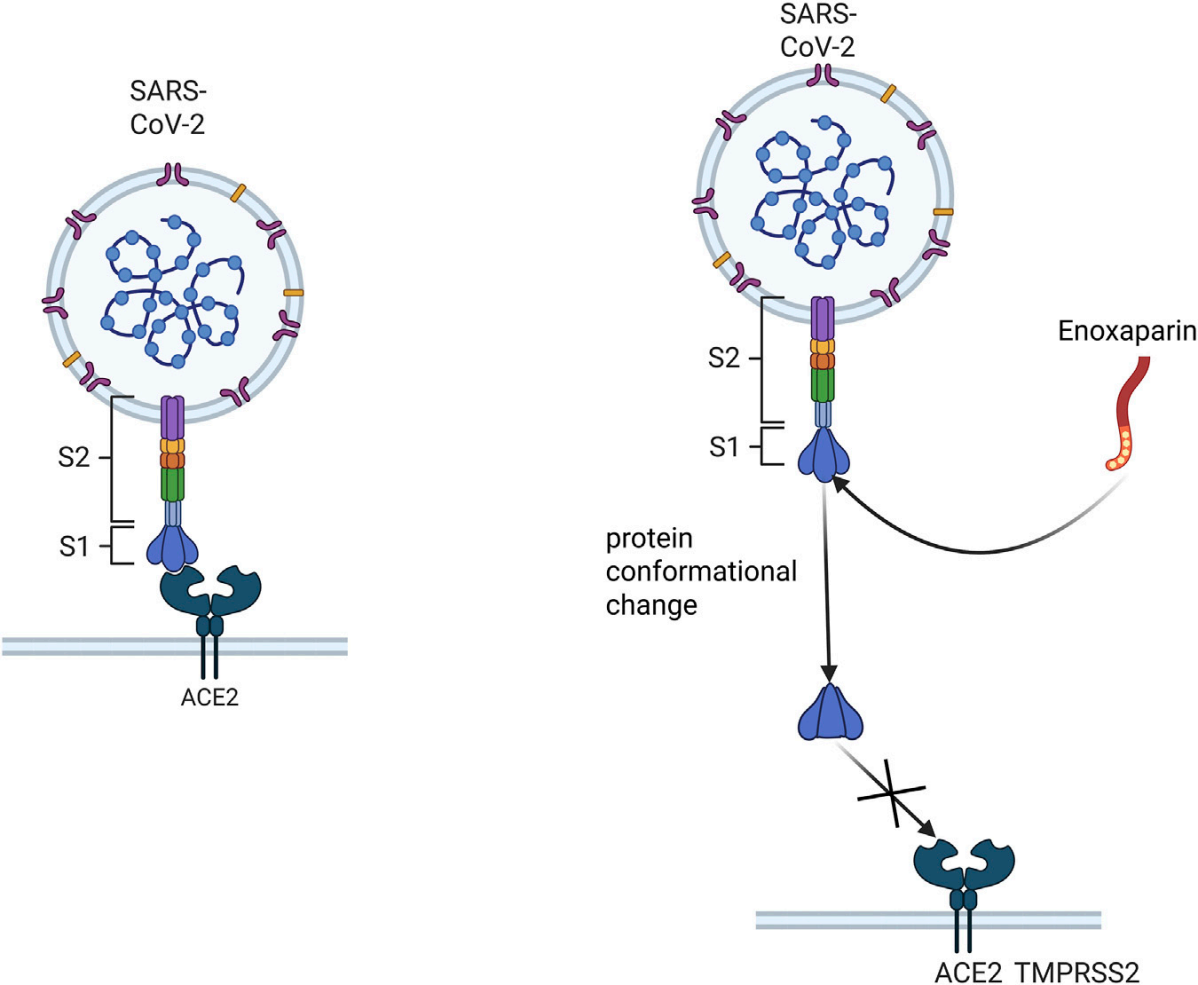
Schematic representation of enoxaparin inhibition of viral binding to host cell receptors. This figure demonstrates that enoxaparin, a LMWH, inhibits viral invasion by binding to the spiking protein receptor binding domain (S1 RBD) of the SARS- CoV-2 virus, thus preventing the virus from binding to the host cell surface receptor ACE2.

LMWH inhibits viral invasion by binding to viral surface glycoproteins and blocking viral binding to host cell receptors. For example, enoxaparin is able to bind to the spike protein receptor-binding domain (S1 RBD) of SARS-CoV-2, and this interaction leads to a conformational change in the viral S protein (Figure 3), which prevents or reduces viral binding to the host cell surface receptor ACE2 (Mycroft-West et al., 2020). LMWH is also able to inhibit viral attachment and invasion by competitively blocking the binding site of viruses to HSPGs on the host cell surface through the interaction with viral surface proteins. This effect is achieved through electrostatic interactions between LMWH and basic amino acid residues on the viral surface (Cagno et al., 2019). The specific antiviral mechanism of LMWH has not been fully characterized, and more studies are needed to further elucidate its mechanism.

#### 2.5.2 Antitumor mechanism

The formation of new blood vessels is necessary for the growth and metastasis of tumor cells. It provides oxygen and nutrients to tumors and is also a mediator of tumor cell metastasis. LMWH can prevent angiogenesis by inhibiting the binding of vascular endothelial growth factor (VEGF) and basic fibroblast growth factor (bFGF) to its receptor (Li et al., 2015), interfere with the adhesion of tumor cells to endothelial cells as well as the formation of the tumor cell–platelet–neutrophil complex to inhibit tumor metastasis, and enhance the NK cell-mediated cytolytic activity of tumor cells, as well as reducing tumor stromal degradation by inhibiting heparanase activity to inhibit tumor invasion and metastasis. In addition, LMWH may affect the CXCL12/CXCR4 signaling pathway to further regulate the migration and invasive behavior of tumor cells (Du, 2019).

Mechanisms of prevention and treatment of anemia: a case–control study indicated that LMWH may affect iron metabolism by regulating serum heparin-binding protein levels, thus exerting some effects on anemia (Azab et al., 2016). However, the study did not provide direct data on the therapeutic effect of LMWH on anemia, and more studies are needed to further elucidate and confirm its specific mechanism of action and effect on anemia.

#### 2.5.3 Anti-fibrotic mechanism

TGF-β1 is a potent pro-fibrotic factor that promotes the proliferation and differentiation of fibroblasts and increases the synthesis of extracellular matrix, which leads to tissue fibrosis (Kim et al., 2018). HGF is a potent anti-fibrotic cytokine that inhibits TGF-β1 through multiple mechanisms, whereas LMWH can indirectly exert anti-fibrotic effects by promoting HGF production, which reduces TGF-β1-induced α-SMA and type-III collagen expression while increasing matrix metalloproteinase (MMP)-2 expression and promoting apoptosis of myofibroblasts, thus reversing fibroproliferative disorders with excessive wound healing (Feng et al., 2023). HGF was able to increase the phosphorylation of AMPK and ACC in tendon fibroblasts and inhibit TGF-β1-induced myofibroblast differentiation in an AMPK signaling pathway-dependent manner (Cui et al., 2013). Hepatic stellate cells (HSCs) are cells that play a key role in the process of liver fibrosis, and LMWHs have an inhibitory effect on the ERK signaling pathway and phosphorylation levels, which reduces the activity of activator protein-1 (AP-1), thereby decreasing the proliferation of HSCs and avoiding the further progression of fibrosis (Feng et al., 2023). The potential of LMWHs in preventing or slowing the progression of fibrosis holds great promise for the treatment of these chronic diseases and offers new hope.

LMWH exerts its biological effects by binding to a variety of biomolecules through its unique structural properties. These interactions are primarily mediated by electrostatic forces, hydrogen bonding, and steric hindrance, with its sulfated structure playing a pivotal role. Specifically, LMWH comprises a highly sulfated branched linear polysaccharide chain that carries a significant negative charge, which is primarily derived from sulfate and carboxylic acid groups. This negative charge enables strong interactions with positively charged amino acids (such as lysine, arginine, or histidine) or specific cationic groups on protein receptors, thereby facilitating binding to a broad spectrum of proteins to exert its biological functions.

Moreover, LMWH has a shorter glycosaminoglycan chain than UFH, which reduces the likelihood of nonspecific binding to nontarget proteins. This feature allows LMWH to selectively bind to specific proteins, thereby enhancing its targeted biological activity. Additionally, as a low-molecular-weight mixture derived from heparin, LMWH can exhibit diverse chain structures and varying degrees of sulfation depending on the specific depolymerization techniques and methods employed (e.g., chemical vs. enzymatic). These structural variations may lead to distinct mechanisms of action and biological effects, thus potentially enabling research workers to identify novel therapeutic mechanisms that warrant further investigation.

### 2.6 Pharmacokinetic profile

As structurally diverse mixtures produced through different preparation methods, LMWH has a lower molecular weight (typically 2–8 kDa) than UFH (12–15 kDa) and a more homogeneous structure than UFH, which gives it a relatively well-defined pharmacokinetic profile. The pharmacokinetic profile of LMWH is as follows:

#### 2.6.1 Bioavailability

The bioavailability of LMWH (approximately 90%) (Bara et al., 1985; Pedersen et al., 1991) was significantly higher than that of UFH (ranges from 30% at lower doses to as much as 70% at higher doses) (Hirish and Raschke, 2004). The enhanced bioavailability of LMWH can be attributed to its lower molecular weight resulting in prolonged half-life, which enables more efficient absorption following subcutaneous injection. In contrast, the higher molecular weight of UFH leads to its less efficient absorption after subcutaneous administration (Fareed et al., 2003).

#### 2.6.2 Linear pharmacokinetics

Unlike UFH, LMWH is shown to have linear pharmacokinetics (Frydman et al., 1988), with anti-Xa (and in some cases anti-IIa) plasma concentrations proportional to the dose over a range of doses, and the volume of distribution and clearance remains constant as the dose increases (Frydman, 1996; Fareed et al., 2003).

#### 2.6.3 Volume of distribution

The volume of distribution of LMWH is close to the blood volume (Pedersen et al., 1991), and they are metabolized partly by desulfation and depolymerization, but for doses that are used to prevent VTE, urinary excretion of anti-Xa activity varies between 5% and 10% of the injected dose (Weitz, 1997). The volume of distribution for enoxaparin’s anti-factor Xa activity is approximately 4.3 L. For dalteparin, the volume of distribution for anti-Xa activity ranges between 40 mL/kg and 60 mL/kg (Jupalli and Iqbal, 2025; Thornton and Parmar, 2025).

#### 2.6.4 Metabolism and excretion

LMWH is mainly excreted *via* the kidneys. In patients with normal renal function, approximately 40%–60% of enoxaparin is cleared by the kidneys and approximately 80% of tinzaparin is excreted by the kidneys. The elimination of nonrenal clearance varies among different LMWHs. The elimination of LMWHs with a low average molecular weight, such as enoxaparin (4,500 Da), is more dependent on intact renal function than that of LMWHs with a high average molecular weight, such as tinzaparin (6,500 Da), resulting in them having different apparent elimination half-lives and bioavailability differences (Johansen and Balchen, 2013).

#### 2.6.5 Half-life

The plasma half-life of LMWH is approximately 3–7 h. Different types of LMWHs have different half-lives. For example, enoxaparin has a half-life of approximately 4 h, dalteparin has approximately 2 h, tinzaparin has approximately 3.5 h, and so on (Bratt et al., 1986; Bara and Samama, 1988), and coagulation generally returns to normal at approximately 12–24 h after discontinuation (Bara and Samama, 1988).

#### 2.6.6 Anticoagulant activity

LMWH enhances the inhibitory effect of ATIII on factor Xa mainly by binding to ATIII, and it weakly inhibits factor IIa (prothrombin), resulting in stronger antithrombotic and weaker anticoagulant effects and a reduced risk of bleeding (Rezaie and Giri, 2020).

#### 2.6.7 Pharmacokinetics in special populations

LMWH does not significantly cross the placenta in pregnant women (Thomson et al., 1998), and the excretion profile is only slightly altered at the prophylactic dose in patients with severe renal insufficiency (endogenous creatinine clearance less than 15 mL/min) (Frydman, 1996).

The well-defined pharmacokinetics of LMWH make their clinical use more reliable and convenient.

## 3 Clinical application and safety

LMWHs exert their anticoagulant effects by inhibiting the final common pathway of the coagulation cascade reaction, and they have a long plasma half-life and low bleeding risk, making them widely used in a variety of thrombotic situations. In addition to anticoagulation, LMWHs have mechanisms of action such as antiviral effects, inhibition of angiogenesis, inhibition of cell adhesion, prevention of glycocalyx shedding, protection of endothelial cells, and anti-inflammatory and anti-fibrotic effects, making them important in a wide range of clinical situations.

### 3.1 Thrombosis

A thrombus is an abnormal clot formed by blood within a blood vessel, which occurs as a result of slow blood flow, damage to the endothelium of the blood vessel, or a state of hypercoagulability of the blood. Thrombosis is a key pathological process leading to a variety of serious cardiovascular events such as myocardial infarction, acute ischemic stroke, and DVT (Srikanth and Ambrose, 2012; Heo et al., 2020). Therefore, an in-depth understanding of the mechanisms of thrombosis and its associated therapeutic strategies is essential to improve the prevention and treatment of thrombotic diseases.

Thrombosis is a complex process involving multiple factors and mechanisms. When the endothelium of a blood vessel is damaged, it exposes collagen, which activates platelets and clotting factors, triggering thrombosis. Platelets are activated and aggregate on the surface of damaged vessels, forming platelet thrombi. Meanwhile, coagulation factors convert fibrinogen to fibrin through a cascade reaction to build a stable thrombus structure. Tissue factor (TF) acts as an initiator of the extrinsic coagulation pathway and binds to factor VII to activate subsequent coagulation factors. Inflammatory and immune responses are also involved, with inflammatory cells expressing TF and activating platelets and coagulation factors by releasing proteases. Hemodynamic changes, such as slowing of blood flow or vortex formation, promote platelet and coagulation factors deposition at the site of injury, further promoting thrombosis. Fibrin formation and stability are critical to the structure and persistence of the thrombus and are influenced by the regulation of thrombin activity and the fibrinolytic system. In addition, impairment of the anticoagulant system can lead to an increased risk of thrombosis.

In addition, there are several diseases and specific conditions such as cancer (Frere, 2022; Kaptein, 2022; Girardi et al., 2023; Gulati and Eckman, 2023; Masini, 2023; Cohen et al., 2024; Cutsem, 2024; Li, 2024; Linder et al., 2024; Pantazi, 2024), antiphospholipid syndrome (Shen, 2022; Jo et al., 2023; Bitsadze et al., 2024), inflammation (Stark, 2021), virus (Alsagaff et al., 2022), severe blunt injuries (Chan et al., 2024), atherosclerosis (Asada et al., 2020), and postoperative orthopedic surgery (Wang et al., 2023) that can lead to thrombus formation.

#### 3.1.1 Venous thromboembolism

VTE is a group of disorders in which blood abnormally clots in the venous system to form blood clots, and it consists of two main types: DVT and PE.

DVT is a blood clot that forms when blood clots abnormally in the deep venous system. This type of thrombus usually occurs in the deep veins of the legs, especially in the calf muscle venous plexus of the calves (Kesieme et al., 2011; Waheed et al., 2025), but it can also occur in other parts of the body (Chan and Weitz, 2020). DVT is a common vascular disease that can lead to serious health problems, especially when the clot dislodges and moves with the blood flow to the lungs, where it can cause PE (Turetz et al., 2018), a potentially life-threatening condition. Meanwhile, up to 50% of DVT patients develop post-thrombotic syndrome (Wang, 2023), which manifests as chronic leg discomfort, edema, skin pigmentation, and venous ulcers, all of which severely affect patients’ quality of life.

The formation of DVT is a complex pathologic process that involves the Virchow triad: blood hypercoagulability, venous stasis, and vascular injury. In this process, the hypercoagulable state of the blood increases the likelihood of thrombosis, venous stasis slows down blood flow, and vascular injury initiates the coagulation cascade, and these three factors interact with each other to promote thrombus formation in the deep veins (Stone et al., 2017).

Treatment of DVT usually includes anticoagulation, intervention, and surgery. Anticoagulation is the cornerstone of DVT treatment, and the commonly used anticoagulants include heparin, LMWH, warfarin, rivaroxaban, apixaban, edoxaban, and dabigatran (Nisio, 2016). Interventional therapies include catheter-directed thrombolysis (CDT), pharmacomechanical thrombolysis (PMT), and venous stent implantation (Siciliano, 2024). In some cases, such as massive (PE) or extensive DVT, thrombolytic therapy is considered. In patients with contraindications to anticoagulation, inferior vena cava filters are an option, with retrievable filters being preferred (Streiff et al., 2016). Overall, the treatment of DVT needs to be customized to the patient and the characteristics of the thrombus to ensure the safety and efficacy of the treatment.

In the anticoagulation of DVT, LMWH is an anticoagulant with favorable efficacy (The Columbus Investigators, 1997; Nicholson et al., 2020), and it is widely used in clinical practice (Ortel et al., 2020).

In terms of efficacy, a study compared the effects of UFH and LMWH on thrombus resolution and recurrent thromboembolism in patients with deep vein thrombosis. Of the patients receiving UFH, 40.2 percent (129 of 321) had thrombus regression, as compared with 53.4 percent (175 of 328) of patients receiving reviparin twice daily and 53.5 percent (167 of 312) of the patients receiving reviparin once daily. With regard to thrombus regression, reviparin administered twice daily was significantly more effective than UFH (relative risk of thrombus regression, 1.28; 97.5 percent confidence interval, 1.08–1.52), as was reviparin administered once daily (relative risk, 1.29; 97.5 percent confidence interval, 1.08–1.53). Mortality and the frequency of episodes of major bleeding were similar in the three groups (Caldeira, 2014).

In terms of ease of administration, LMWH requires subcutaneous injection, which, when compared to UFH requiring intravenous infusion, offers a more convenient route of administration. However, LMWH possesses a shorter half-life than DOACs, necessitating more frequent dosing (Frcpa, 2014). Furthermore, the absence of an oral dosage form for LMWH, in contrast to DOACs, may potentially affect patient adherence (Zhu et al., 2024).

In terms of safety, LMWH has less incidence of heparin-induced thrombocytopenia (HIT) than UFH (Martel et al., 2005). In terms of use in special populations, the use of LMWH in pregnant women is considered safe, which makes it the preferred choice for the treatment of VTE during pregnancy (Frcpa, 2014). In patients with mechanical heart valves, the use of LMWH is not yet approved; however, it is considered an effective interim treatment option in some guidelines (Caldeira et al., 2014). Its use in patients with renal insufficiency needs to be approached with caution as LMWH clearance is mainly through the kidneys (Nutescu, 2008).

In the anticoagulation therapy of deep vein thrombosis, due to the wide variety of anticoagulant drugs and their different action characteristics, it is necessary to adjust the treatment of anticoagulant drug types and dosages according to the patient’s individual differences (including genetic characteristics, pathophysiological status, and disease phenotypes), so standardized, rationalized, and individualized anticoagulation therapy is crucial, and the individualized anticoagulation therapy needs to be combined with the pathophysiological status of the patient and the evidence-based therapeutic targets. Personalized anticoagulation therapy requires a comprehensive assessment of the characteristics of anticoagulant drugs and their individualized application in order to improve the safety and effectiveness of anticoagulation therapy.

PE is a clinical and pathophysiological syndrome in which blood forms a thrombus in the deep veins and then embolizes and enters the pulmonary arteries and their branches with the blood flow, thus obstructing the pulmonary circulation. Common symptoms include dyspnea, chest pain, cough, and hemoptysis.

The formation of pulmonary embolism is also associated with three elements of thrombosis, which together result in the formation of thrombus by abnormal coagulation of blood in the veins (Kushner et al., 2024). Over time, these thrombi may be partially or completely dislodged hemodynamically and become emboli, and they then travel with the circulation and eventually reach the pulmonary arteries or their branches, causing obstruction (Turetz et al., 2018).

Anticoagulation is used as a basis in the treatment of pulmonary embolism, with the use of warfarin, LMWH, or direct oral anticoagulants to prevent recurrence, particularly rethrombosis (Stevens et al., 2024). In high-risk patients, thrombolytic therapy or mechanical thrombolysis may be required to rapidly restore pulmonary circulation (Lauder et al., 2023). Special populations, such as obese individuals, patients with hepatic and renal insufficiency, the elderly, oncology patients, and children, require individualized anticoagulation regimens. The entire treatment process needs to be individualized according to the patient’s specific situation and the severity of the disease to ensure the best therapeutic outcome.

UFH is the traditional treatment of choice for pulmonary embolism (Aday and Beckman, 2020). However, due to the many limitations of UFH, research workers have developed and tested new LMWHs in several clinical trials, and studies have demonstrated their effectiveness in treating patients with PE (LMWH has shown similar efficacy and safety to UFH in the treatment of PE. In the COLUMBUS trial, 271 patients with PE were treated, and the results showed that the recurrence rates were 5.9% in the LMWH group and 6.0% in the UFH group, with mortality rates of 2.2% and 0%, respectively. In the THÉSÉE trial, 612 patients with symptomatic PE were treated, and the event rates within 8 days (including recurrent VTE, major bleeding, or death) were 3.0% in the LMWH group and 2.9% in the UFH group, whereas the event rates within 90 days were 5.9% in the LMWH group and 7.1% in the UFH group. In a study, 200 patients with high-probability pulmonary perfusion scan results were treated, and there were no recurrences in the LMWH group, whereas the UFH group had seven new events (four of which were PE), with mortality rates of 6.2% and 8.7%, respectively.) (Ageno and Huisman, 2000).

In addition, LMWH demonstrated better therapeutic efficacy than UFH in the treatment of acute pulmonary embolism by decreasing pulmonary artery intimal hyperplasia, decreasing PDGF-b expression, and ameliorating the inflammatory response and thickening of the pulmonary artery wall (Byrne et al., 2017). As DVT pulmonary embolism is two different manifestations of the same condition, LMWH has similar efficacy, safety, ease of administration, and use in special populations as in DVT.

#### 3.1.2 Cancer-related thrombosis

The mechanisms of cancer-associated thrombosis are complex and multifaceted, involving the interaction of direct and indirect mechanisms. Direct mechanisms include activation of the coagulation cascade by the tissue factor expressed by cancer cells, promotion of coagulation complex formation by microparticles carrying TF and phosphatidylserine released by cancer cells, and direct platelet activation by podoplanin (PDPN) *via* the C-type lectin-like receptor 2 (CLEC-2) receptor. In addition, platelet agonists secreted by cancer cells such as ADP and thrombin further promote platelet activation. Indirect mechanisms involve endothelial cell and platelet activation induced by inflammatory cytokines, neutrophil extracellular traps released by neutrophils to promote platelet aggregation and fibrin deposition, and disturbed blood flow due to the interaction of cancer cells with platelets and endothelial cells. In addition, cancer patients often develop a hypercoagulable state, which is related to cancer treatment, individual patient factors, and the nature of cancer itself. Together, these mechanisms lead to an increased risk of thrombosis in cancer patients (Abdol Razak et al., 2018; Halsema and Mcmahon, 2023).

In a large United Kingdom study, the annual incidence of VTE in cancer patients was 13.9 cases/1,000 person-years, compared with only 1.85 cases/1,000 person-years in the general population (Abbas et al., 2025). In another study, the incidence of arterial thrombosis within 6 months of diagnosis was 4.7% in cancer patients compared to 2.2% in non-cancer patients (Navi et al., 2017).

LMWH shows some advantages in the treatment of cancer-related thrombosis; for example, it significantly reduces the risk of recurrence of cancer-related thrombosis compared to vitamin K antagonists (the relative risk for low-molecular-weight heparin was 0.51 with a 95% confidence interval of 0.35–0.74) (Akl et al., 2008). LMWH has high utility in cancer patients, does not require monitoring, and does not have a narrow therapeutic window (van den Broek et al., 2022). LMWH offers greater flexibility than warfarin and has been shown to have a positive impact on the overall quality of life (Farge et al., 2018). Long-term LMWH therapy has shown efficacy in the treatment of VTE in patients with advanced cancer, and current treatment guidelines recommend LMWH as a preferred agent for initial events and secondary prevention in cancer patients (Nishioka and Goodin, 2007).

However, there are some uncertainties associated with LMWH in therapy. Although there is evidence that LMWHs are effective in reducing VTE in patients with certain specific types of cancer, the optimal dose, duration, and specific patient populations still need to be further defined. The results of studies on the survival benefit of LMWH are controversial, and much uncertainty remains regarding the treatment of cancer-related thrombosis and the effects of LMWH on tumor biology. The evidence supporting the use of LMWH in cancer patients comes from a limited number of relatively small clinical studies, which limits the full understanding of the effects of LMWH.

#### 3.1.3 Antiphospholipid syndrome-associated thrombosis

Antiphospholipid syndrome (APS) is an autoimmune disorder characterized by the persistent presence of antiphospholipid antibodies, which bind to phospholipids and phospholipid–protein complexes, specifically interacting with β2-glycoprotein I (β2GPⅠ), a process that activates endothelial cells, the complement system, platelets, and neutrophils, and promotes clot formation. These antibodies are capable of converting the closed, non-immunogenic β2GPⅠ into an open, immunogenic form, which in turn promotes blood clotting. Furthermore, antiphospholipid antibodies may further exacerbate the hypercoagulable state by affecting the function of the anticoagulant and fibrinolytic systems, such as disrupting protein C activation, as well as affecting the degradation of fibrin (Arcilla and Zubair, 2025). *In vivo*, the presence of these antibodies may lead to platelet aggregation and vascular obstruction; however, thrombocytopenia may also be observed in these patients (Van de Vondel et al., 2023). APS increases the risk of thrombosis by affecting multiple coagulation and anticoagulation pathways.

LMWH plays multiple roles in the treatment of APS; it not only reduces the risk of thrombosis through its anticoagulant effect but also has an anti-inflammatory effect that attenuates the inflammatory response caused by antiphospholipid antibodies (Ambati et al., 2023). In APS patients with recurrent thrombosis, LMWH can be a therapeutic option to reduce thrombotic events (Bitsadze et al., 2024). In addition, prophylactic dosage use of LMWH is recommended during pregnancy to reduce the risk of pregnancy complications, and it can further improve pregnancy outcomes when combined with aspirin (Tong et al., 2023). These dual anticoagulant and anti-inflammatory actions render LMWH a crucial treatment modality for patients with APS, particularly those at risk of pregnancy complications.

#### 3.1.4 Blood clots in special populations

Pregnant women face a higher risk of thrombosis during pregnancy due to uterine enlargement, hypercoagulable state of the blood, hemodynamic changes, decreased activity, vascular wall damage, genetic susceptibility to thrombosis, and pregnancy complications (James, 2009; Alsheef et al., 2020; Maughan et al., 2022; Merz et al., 2025; Mojaddedi et al., 2025). Studies show that pregnant women have 4–5 times the risk of VTE as women of the same age in nonpregnant women (Grouzi et al., 2022). In addition, thrombophilia may lead to a range of pregnancy complications, including preeclampsia, fetal dysplasia, oligohydramnios, placenta previa, toxemia of pregnancy, HELLP syndrome, cervical insufficiency, and gestational hypertension (Kujovich, 2004; Brenner and Aharon, 2007; Tsikouras et al., 2018; Mihai et al., 2023). LMWH has demonstrated significant benefits in pregnancy-associated thrombosis, including acting as a potent anticoagulant to reduce pregnancy complication risk (León-García et al., 2024), ensuring maternal and infant safety, increasing live birth rates (women who received treatment had a higher rate of live births than women who did not receive treatment, 71.4% vs. 28.6%, and there were no serious maternal or fetal complications in the treatment group), reducing the risk of recurrent VTE (Eck et al., 2019), decreasing bleeding events, and contributing to full-term delivery of infants (León-García et al., 2024). In addition, the use of LMWH offers the possibility of pregnancy in women with a history of Budd–Chiari syndrome and/or portal vein thrombosis under multidisciplinary monitoring and management (Wiegers et al., 2022). In terms of safety, LMWH is unable to cross the placenta, so it does not pose an immediate risk to the fetus (Galambosi, 2012; Zullino, 2022).

Obesity, particularly central (visceral) obesity, is associated with an increased incidence and prevalence of arterial and venous thrombotic events. The various mechanisms by which obesity may contribute to thrombosis include the action of the so-called adipocytokines (e.g., leptin and lipocalin) in adipose tissue, increased activity of the coagulation cascade and decreased activity of the fibrinolytic cascade, increased inflammation, increased oxidative stress and endothelial dysfunction, and disturbances in lipid and glucose tolerance associated with the metabolic syndrome (Clark, 2008). Fixed-dose anticoagulant regimens may not provide optimal VTE prophylaxis in obese patients due to their weight and body composition, which significantly affects drug distribution and pharmacokinetics, and therefore, dose adjustments are necessary to optimize efficacy and reduce the risk of VTE in obese patients (Freeman et al., 2010).

Renal insufficiency is a clinical syndrome in which renal function is diminished due to a variety of causes, resulting in disturbances in the body’s excretion of metabolic wastes and regulation of water–electrolyte and acid–base balance, among others. The thrombotic effect of LMWH in patients with renal insufficiency requires special attention because LMWH is mainly excreted through the kidneys, and renal insufficiency may lead to drug accumulation in the body and increase the risk of bleeding (Schmid et al., 2009). In special cases, such as severe renal insufficiency, monitoring of anti-Xa activity may be required to assess the anticoagulant effect of LMWH (Nutescu et al., 2009). When using LMWH in patients with renal insufficiency, the renal function status of patients should be strictly assessed, and the treatment program should be adjusted according to the specific situation to ensure the effectiveness and safety of anticoagulation therapy (Schmid et al., 2009).

### 3.2 Cancer

LMWH influences cancer cell adhesion, angiogenesis, migration, and invasion through diverse mechanisms. Bokas et al. (2020) comprehensively reviewed the mechanisms of action of LMWH in pancreatic cancer therapy, highlighting several key effects. LMWH inhibits tumor angiogenesis and invasion by suppressing heparinase activity and diminishing extracellular matrix degradation. Furthermore, it reduces tumor metastasis by blocking P- and L-selectin and VLA-4/VCAM-1-mediated cellular interactions. LMWH also promotes the release of tissue factor pathway inhibitor (TFPI), thereby impairing tumor neoangiogenesis. In addition, LMWH reverses the immunosuppressive microenvironment by reducing hypoxia and inhibiting VEGF, promoting effector T-cell activity, reducing fibroblast activation and fibrosis, and enhancing drug delivery within the tumor microenvironment. These combined effects promote vascular normalization and reprogram the microenvironment to become more supportive of the immune response, ultimately improving therapeutic efficacy.

In addition to the above mechanisms, LMWH may also reduce tumor metastasis by inhibiting platelet–cancer cell interactions (Mousa et al., 2006). Platelets play a key role in the coagulation process in cancer patients, and there is evidence that platelets act as a bridge connecting cancer cells to the endothelium, enhancing cancer cell adhesion and metastasis (Anvari et al., 2021).

Several clinical trials and meta-analyses have shown that LMWH improves survival in cancer patients (Ma et al., 2020); however, this benefit varies by tumor type, cancer stage, and type of LMWH (Lazo-Langner, 2007). For example, a FAMOUS study showed that the ovarian cancer patients treated with LMWH had a reduction in mortality from 37.5% to 24% at 2 years (Kakkar et al., 2004).

LMWH has a promising application in tumor therapy, and its antitumor, antiangiogenic, and antimetastatic activities make it a promising anticancer drug. However, the pharmacological mechanisms, therapeutic efficacy, and safety profiles of LMWH in oncology require further investigation.

### 3.3 Pregnancy and obstetrics

LMWH has significant clinical value in the field of pregnancy and obstetrics, especially in the treatment of recurrent spontaneous abortion (RSA), fetal growth restriction (FGR), and intrahepatic cholestasis of pregnancy (ICP).

#### 3.3.1 RSA

LMWH has demonstrated efficacy in the treatment of certain patients with RSA, particularly in patients with acquired pre-thrombotic states (PTS) such as antiphospholipid syndrome (APS). The use of LMWH may increase the rate of live births (approximately 30%) in repeat pregnancies and improve pregnancy outcomes (Papadakis, 2019). In addition, the study indicated that enoxaparin was the most effective treatment for recurrent spontaneous abortions with LMWH, significantly increasing the rate of live births (30%–40%) and reducing the risk of preeclampsia (30%–40%), preterm labor (20%–30%), and pregnancy loss (40%–50%) (Huang et al., 2025).

#### 3.3.2 FGR

Through multiple pathways, including ameliorating the hypercoagulable state of blood, reducing blood viscosity, lowering vascular resistance, protecting vascular endothelial cells, and mitigating fibrin deposition in placental villi, LMWH enhances fetal growth and development, ultimately improving neonatal survival rates (Papadakis, 2019; Chen et al., 2024; Xu et al., 2024).

#### 3.3.3 ICP

Although there are no studies directly referring to the role of LMWH in ICP, it has been shown that LMWH alters placental function and thus reduces hyperviscosity and thrombosis, microcirculatory disturbances, hyperlipidemia, and hyperfibrinogenemia in patients with ICP (Papadakis, 2019).

In summary, LMWH has a wide range of applications in pregnancy and obstetrics, especially playing an important role in the treatment of RSA and FGR, and in ICP. However, the use of LMWH should be under the guidance of a physician to ensure the safety of the mother and the fetus.

### 3.4 Emerging and potential applications

LMWH demonstrates expanding clinical utility due to its established mechanisms of action and the potential therapeutic targets derived from these pathways.

Unexplained subfertility is a condition in which the patient’s fertility is significantly lower than normal, but no clear cause is found after routine testing. In contrast to unexplained infertility, subfertility is not a complete inability to conceive but rather a lower chance of getting pregnant or the presence of recurrent pregnancy failure. In assisted reproductive technology (ART), LMWH is used to treat unexplained subfertility and has been associated with increased live birth rates (OR 1.77; 95% confidence interval, 1.07–2.90; 3 RCTs; 386 women) (Holt-Kentwell et al., 2022). Although the use of LMWH in ART shows some potential, the current quality of evidence is very low, so its use in clinical practice requires cautious application, and more high-quality studies are needed to further validate its efficacy and safety.

Neurodegenerative diseases are a group of disorders characterized by progressive degeneration or death of neurons. These diseases usually affect the central nervous system (brain and spinal cord), resulting in a progressive loss of cognitive, motor, and behavioral functions. Neurodegenerative diseases are common in older age-groups, but certain types can also occur in younger populations. LMWH (enoxaparin) has shown significant potential in attenuating coagulation and neuronal damage in a lipopolysaccharide (LPS)-induced C57BL/6J mouse model (Berkowitz et al., 2022). Its neuroprotective and anti-inflammatory effects offer promising applications in the treatment of neurodegenerative diseases and the development of neuroprotective agents. Future studies will further explore the efficacy and mechanism of enoxaparin in neurological diseases and provide more evidence to support its clinical application.

Diabetic nephropathy is a chronic kidney disease caused by diabetes mellitus with a complex pathogenesis, and the main clinical features are persistent increased urinary albumin excretion and progressive decrease in the glomerular filtration rate, which may eventually progress to end-stage renal disease. Several studies have indicated that LMWH shows the potential to reduce proteinuria (Benck et al., 2007), protect renal function (Guo et al., 2001), activate the PPAR signaling pathway, and enhance renal barrier function in diabetic nephropathy (DN) (Zhang, 2024), and these effects may be mediated by direct actions on glomerular filtration properties, modulation of renal hemodynamics under diabetes mellitus, intracellular PPAR activation, and protection of tubular epithelial cells by mechanisms such as glycocalyx preservation, providing new strategies and hope for the treatment of diabetic nephropathy.

COVID-19 is an infectious disease caused by the SARS-CoV-2 coronavirus, whose full name is “Coronavirus Disease 2019.” The virus first appeared in late 2019 and subsequently spread rapidly across the globe, leading to a massive public health crisis. Symptoms of COVID-19 include fever, dry cough, malaise, and shortness of breath, which in severe cases can lead to pneumonia, acute respiratory distress syndrome, multi-organ failure, and even death (Harapan et al., 2020). Due to its high contagiousness, it has a major impact on global health and socio-economics. A study by Eder et al. (2022) found that LMWH administered by inhalation can effectively prevent SARS-CoV-2 virus from attaching to human nasal epithelial cells, thus acting as a method of preventing infection. Inhalation of LMWH *in vivo* was comparable to *in vitro* LMWH application, and no adverse events were observed in study participants. The fact that LMWH is generally available, inexpensive, and easy to apply makes it a suitable candidate for the prevention of SARS-CoV-2 infection.

Ulcerative colitis (UC) is a chronic nonspecific inflammatory bowel disease that primarily affects the mucosa of the large intestine, especially the rectum and colon, and is characterized by recurrent episodes of intestinal inflammation leading to abdominal pain, diarrhea, and blood in the stools, accompanied by a variety of potential complications, including an increased risk of cancer. The disease has an unpredictable course and is currently incapable of being cured, which has a significant impact on patients’ quality of life. Huang et al. (2024) have developed a novel oral nanotherapeutic agent that achieves precise targeting of the inflammatory site and regulates intestinal redox homeostasis by exploiting the specific interaction of LMWH with integrin αM, thus demonstrating significant efficacy for the treatment of UC in *in vitro* and *in vivo* experiments, including reducing inflammation, inhibiting oxidative stress, and protecting the colonic epithelium, thus providing a potential new strategy for precision treatment of UC.

The potential role of LMWH continues to expand its therapeutic scope, extending from the traditional anticoagulant effect to various fields such as antiviral, antitumor, and neuroprotection, showing its multifaceted potential in regulating immune responses, inhibiting tumor growth and metastasis, and ameliorating neurological disorders. These new discoveries provide a new direction for the future research and clinical application of LMWH, and foretell a broad application of LMWH in the field of medicine. These new discoveries provide a new direction for future research and clinical application of LMWH, predicting its broad applicability in medicine.

### 3.5 Safety

The safety challenges associated with LMWH primarily arise from its inherent complexity as a biological agent, encompassing the following: (1) immune reactions stemming from its immunogenicity, (2) manufacturing variations arising from complex production processes, (3) interindividual variability in pharmacokinetic responses resulting from dosing inaccuracies and inadequate monitoring strategies, and (4) drug–drug interaction potentials. These multifaceted characteristics collectively pose significant safety challenges in clinical practice.

#### 3.5.1 Bleeding risks and management strategies in LMWH

LMWH has a lower bleeding risk relative to UFH due to its selective inhibition of factor Xa, but risk management in special groups (e.g., patients with renal dysfunction) still requires attention (Weitz, 1997). Monitoring of anti-Xa activity is required for long-term use, especially at high doses or in the presence of other risk factors (e.g., bleeding risk or renal dysfunction), and monitoring is essential to minimize side effects and risks (Jack, 1998; Liu et al., 2023).

#### 3.5.2 Long-term safety analysis of LMWH

##### 3.5.2.1 Reduced bone density

Studies have shown that long-term use of LMWH may lead to a reduction in bone density by interfering with osteoblast function and altering calcium metabolism, especially in the elderly population (Gajic-Veljanoski et al., 2016). A study looked at 86 patients on long-term LMWH for 2 years. The results showed an average decrease in bone density of approximately 3.1% and 4.8% in these patients (Wawrzyńska et al., 2003). This reduction in BMD, if it progresses to osteoporosis, significantly increases the risk of fractures, especially in the hip, spine, and wrist. Fractures may not only lead to physical problems such as chronic pain, shortened height, hunchback, and limited mobility but may also cause other health problems such as pneumonia and blood clots due to prolonged bed rest.

##### 3.5.2.2 Renal impairment

Long-term use of LMWH can adversely affect able renal function, especially in patients with preexisting problems with renal function itself (Mismetti and Laporte, 2001). Renal insufficiency can lead to decreased drug clearance of LMWH, increasing the risk of its accumulation, which may further burden renal function (Lim et al., 2006). In the study by Feng et al. (2006), the clearance rate of enoxaparin was reduced by 22% in patients with end-stage renal disease (ESRD).

##### 3.5.2.3 Immune response

LMWH may lead to immune system reactions such as HIT (Liu et al., 2023), an immune-mediated complication usually associated with UFH use, but LMWH may also cause this problem (Mammen, 1999).

A study evaluating the clinical characteristics and pharmacovigilance of HIT associated with LMWH found that LMWHs had significantly higher rates of adverse event reporting in the FDA Adverse Event Reporting System (FAERS) database, with tinzaparin having a higher proportionate reporting ratio (PRR) and reporting odds ratio (ROR) than other LMWHs, suggesting that it is more likely to cause HIT. The overall number of the reported AEs of enoxaparin, dalteparin, and tinzaparin was 242, 34, and 30, respectively. Most AEs of the three LMWHs occurred in people aged over 60 years, ranging between 50% and 70%. The proportion of AEs in male and female patients is similar. Among the AEs of enoxaparin and tinzaparin, male patients accounted for more than 50%, and in the AEs of dalteparin, female patients accounted for more than 50%. The LMWH with the highest proportion of deaths among the outcomes of AEs was tinzaparin, accounting for 36.67%, whereas the lowest was enoxaparin, accounting for 21.90%. Enoxaparin had the highest proportion of reported cases in the United States, accounting for 38.43%.

The study also noted evidence of LMWH-induced thrombocytopenia in 43 patients, with a median onset time of 8 days, and almost half of the events were caused by enoxaparin. In addition, patients with a history of diabetes or surgery appear to be more likely to develop HIT. Although LMWH-induced thrombocytopenia is rare, it does represent a serious problem, especially in patients with an increased risk of diabetes or a history of surgery. Timely recognition and management is essential for the safe use of LMWH (Liu et al., 2023).

Although LMWH is associated with certain bleeding risks and safety concerns, particularly the risk of adverse reactions, it remains a key therapeutic tool in the prevention and treatment of thromboembolic diseases due to its excellent antithrombotic effect, high bioavailability, ease of administration, and wide clinical application. These concerns can be substantially reduced through enforcing strict dosage control and regular monitoring. Meanwhile, through the implementation of strict dosage control, regular monitoring, individualized treatment regimens, management of drug interactions, training in injection techniques, patient education, and the preparation of alternative therapeutic regimens, its safety concerns and the risk of adverse reactions can be significantly reduced to ensure that patients can receive safe and effective treatment.

## 4 Discussion

Since its introduction, LMWH has rapidly become a major therapeutic option for thromboembolic diseases due to its superior anticoagulant activity and lower bleeding risk. However, there are still many shortcomings in the clinical application of LMWH.

First, in terms of individualized treatment, the current dosage adjustment of LMWH is mainly based on body weight, renal function assessment, and monitoring of anti-Xa activity, which fails to adequately take into account individual differences in patients’ genotypes, coagulation status, and comorbidities. This standardized approach to dosing may lead to inadequate efficacy or increased risk of bleeding in some patients, especially in patients with renal insufficiency.

Individualized medicine is a key direction to improve the safety and efficacy of LMWH clinical application. Combined with precision medicine technology, the dosage and regimen of medication for patients can be optimized through genetic testing and proteomics analysis. For example, patients with defective antithrombin III function identified through genetic testing can have their LMWH dosage increased, whereas patients with elevated heparinase levels of heparinases found through proteomics may need to have their LMWH dosage adjusted or other anticoagulant drugs selected to avoid excessive degradation of LMWH in the body, which can lead to a decrease in therapeutic efficacy. These personalized adjustments can effectively improve efficacy, reduce adverse effects, and thus optimize treatment outcomes.

Technological innovation is also an important driver for the development of LMWH. For example, analyzing patient data using artificial intelligence (AI) and big data enables dynamic optimization of medication regimens while allowing real-time monitoring of patients’ coagulation status with wearable devices to develop dynamically optimized, personalized anticoagulation regimens. Beyond clinical applications, these technologies also advance fundamental research by investigating the mechanisms of LMWH in complex pathological environments. Specifically, machine learning algorithms can be employed to predict the efficacy of LMWH in different tumor microenvironments.

Second, the safety challenges associated with LMWH stem from its inherent complexity as a biological agent, encompassing the following: (1) immune reactions associated with its immunogenicity, (2) manufacturing variations arising from complex production processes, (3) interindividual variability in pharmacokinetic responses requiring precise dosing and therapeutic monitoring, and (4) drug–drug interaction potentials with concomitant medications. These multifaceted characteristics collectively pose significant safety challenges in clinical practice. Whereas subcutaneous LMWH is convenient for acute-phase treatment, poor adherence in patients requiring long-term anticoagulation (e.g., those with cancer-related thrombosis or chronic cardiovascular disease) compromises therapeutic efficacy and may increase the risk of thrombotic recurrence. Future studies should focus on developing novel drug delivery systems, including oral formulations, transdermal patches, and inhalation formulations, which may enhance adherence and efficacy. Furthermore, nanotechnology-incorporated extended-release formulations, potentially reducing dosing frequency through controlled drug release mechanisms, represent another promising avenue for research.

In patients who have been using LMWH for a long time, special attention should be paid to issues of osteoporosis and renal dysfunction. To mitigate fracture risk, combination therapies, such as alternating or combining LMWH with other anticoagulants such as aspirin or warfarin, can be considered to minimize the adverse effects of any single agent. Future research may explore combining LMWH with medications that counteract its side effects, such as calcium supplements, vitamin D, furosemide, spironolactone, or mannitol. Additionally, consideration should be given to whether the long-term anti-inflammatory effects of LMWH might suppress the immune system’s surveillance function against tumors and whether changes in the volume of distribution of LMWH could lead to drug accumulation in adipose tissue, potentially triggering chronic inflammation. In clinical practice, these potential issues should be closely monitored, and individualized treatment plans should be developed based on the specific circumstances of the patient to ensure the safety and effectiveness of the treatment.

The potential of LMWH in antiviral, antitumor, and neurodegenerative disease therapies is gradually being discovered, but large-scale clinical trial validation is lacking. Taking COVID-19 as an example, LMWH can effectively reduce the risk of viral invasion by blocking the binding of viral glycoproteins to host receptors. This mechanism may be equally applicable in other viral infections, such as influenza and dengue fever. In addition, the anti-inflammatory and neuroprotective effects of LMWH suggest its use in the treatment of neurodegenerative diseases, such as Alzheimer’s disease, Parkinson’s disease, amyotrophic lateral sclerosis, and Huntington’s disease. Future studies could further validate the efficacy of these emerging indications through multicenter randomized controlled trials (RCTs). By expanding the indications, LMWH is expected to reach more patients globally and bring new hope for the comprehensive treatment of complex diseases.

The chemical modification of LMWH has great potential, and the diversity of its structure provides rich targets for chemical modification. The chemical modifications of LMWH mainly focus on the following areas:

Nonreducing end (NRE) and reducing end (RE): the nonreducing end and reducing end of LMWH undergo significant changes during the chemical depolymerization process. For example, selective cleavage of UFH can be achieved through oxidation with sodium periodate and reduction with sodium borohydride, resulting in LMWH with lower anticoagulant activity than the parent UFH molecules. This chemical modification not only alters the anticoagulant activity of LMWH but also lays the foundation for its further functional expansion.

Backbone structure: the backbone structure of LMWH can be modified through various chemical methods. For instance, acylation modification is a common approach. Reactions such as acetylation or butyrylation can significantly change the biological activity of LMWH. Studies have shown that LMWH after acetylation (ALMWH) has a significantly reduced anticoagulant activity, but its activity against tumor cell proliferation and invasion is significantly enhanced. This modification affects the interaction of LMWH with biomolecules by altering its degree of sulfation and charge distribution.

Hydroxyl and carboxyl group modifications: the hydroxyl and carboxyl groups in LMWH molecules are also important sites for modification. For example, using N,N′-Dicyclohexylcarbodiimide (DCC) and 4-dimethylaminopyridine (DMAP) as catalysts, the hydroxyl groups of LMWH can be acetylated, whereas the carboxyl groups can be modified with acylurea. This type of modification not only reduces the anticoagulant activity of LMWH but also enhances its antitumor activity, demonstrating the significant potential of chemical modification in regulating the functions of LMWH.

Oxidative modification: oxidative modification is another important method of chemical modification. For example, hydrogen peroxide oxidation can degrade UFH to generate LMWH with different molecular weights and sulfation degrees. Oxidative modification can not only alter the molecular weight distribution of LMWH but also regulate its bioactivity, enabling it to exhibit different properties in anticoagulation, antitumor applications, and other areas.

LMWH has broad application prospects in regulating its biological activity through chemical modification. By chemically modifying various parts of LMWH, such as its nonreducing end, reducing end, main chain structure, hydroxyl groups, and carboxyl groups, its anticoagulant activity, antitumor activity, and other biological functions can be significantly altered. These modifications not only offer possibilities for developing new drugs but also provide important theoretical insights into the structure–function relationship of LMWH.

This review systematically summarizes the mechanism of action, pharmacokinetic characteristics of LMWH, and its wide range of applications in thromboprophylaxis and therapy, especially its advantages in special populations (e.g., tumor patients and pregnant women). Meanwhile, the potential of LMWH in anti-inflammation, immunomodulation, and in non-anticoagulant fields such as antitumor and antivirus was also thoroughly explored, providing a reference for the future research and clinical application of LMWH, revealing its potential in thromboprophylaxis, anti-inflammation, immunomodulation, and anticancer therapy, and advancing the understanding of its multifunctionality. Future research should focus on the development of more efficient and safer LMWH derivatives, personalized therapeutic strategies, and the expansion of new indications, especially in antiviral and neuroprotective applications. Meanwhile, the review provides a scientific basis for clinicians to use LMWH in special populations and promotes its broader clinical utilization in clinical treatment.

**FIGURE 4:**
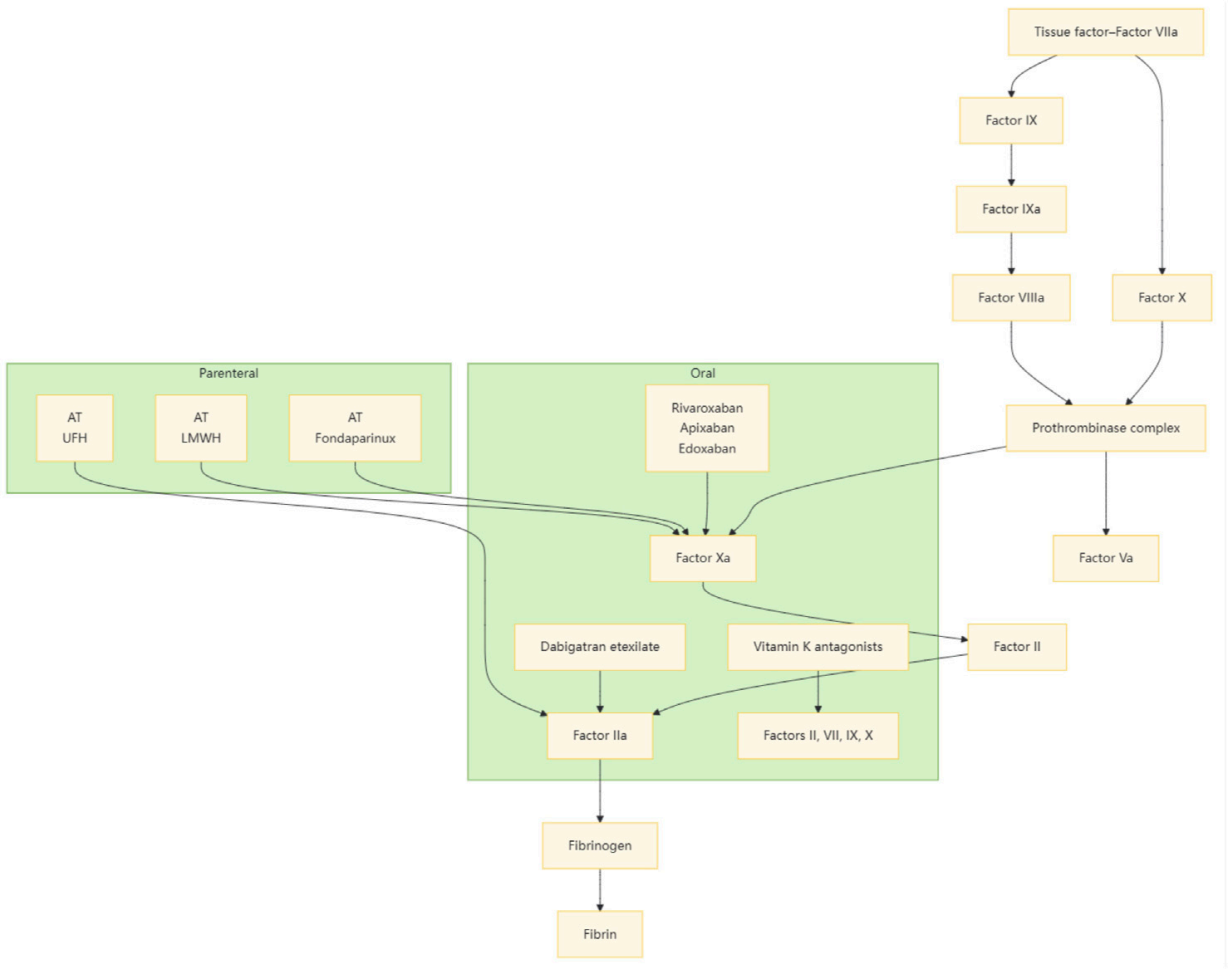
Intravenous and oral anticoagulants inhibit the clotting process by interfering with different stages of clotting factors.

Due to space limitations, in this article, we only offer a concise comparison of the indications and mechanisms of action of LMWH with those of other anticoagulant medications, such as aspirin, warfarin, and UFH (Table 1; Figure 4). Further studies can be conducted to more comprehensively evaluate the status and advantages of LMWH in various therapeutic regimens.

**TABLE 1:** Comparison between anticoagulants.

Medicine Feature	Low-molecular-weight heparin	Aspirin	Warfarin	Unfractionated heparin	Rivaroxaban	Dabigatran etexilate
Mechanism of action	Activates antithrombin III and inhibits factor Xa, thus preventing thrombin formation	Inhibits cyclooxygenase (COX) and reduces thromboxane A2 production, thereby inhibiting platelet aggregation	Inhibits the synthesis of vitamin K-dependent clotting factors (II, VII, IX, and X), prolonging clotting time	Binds to antithrombin, enhancing its inhibition of factors Xa and IIa, thus exerting anticoagulant effects	Selectively inhibits factor Xa, preventing thrombin generation	Directly inhibits thrombin (factor IIa), preventing the conversion of fibrinogen to fibrin
Indications	Deep vein thrombosis (DVT), pulmonary embolism (PE), unstable angina, and non-Q wave myocardial infarction	Prevention of cardiovascular events, transient ischemic attack (TIA), acute myocardial infarction, and stable angina	Atrial fibrillation, heart valve disease, deep vein thrombosis, and pulmonary embolism	Acute coronary syndrome, extracorporeal circulation, and hemodialysis	Deep vein thrombosis, pulmonary embolism, and atrial fibrillation	Atrial fibrillation, acute coronary syndrome, and deep vein thrombosis
Route of administration	Subcutaneous injection	Oral	Oral	Intravenous injection	Oral	Oral
Bleeding risk	Low	Low	High	High	Low	Low
Monitoring	Generally, no routine monitoring is required, but anti-Xa activity can be monitored in special cases	Generally, no routine monitoring is required, but platelet count can be monitored	Requires regular INR monitoring	Requires APTT monitoring	Generally, no routine monitoring is required	Generally, no routine monitoring is required
Half-life	4–5 h	Salicylic acid half-life is 2–3 h	36–42 h	1–2 h	5–13 h	12–17 h
Bioavailability	90%	68%	>90%	Bioavailability of subcutaneous injection is only 30%	66%	6.50%
Renal clearance	Mainly excreted *via* the kidneys; clearance varies slightly depending on the molecular weight	Low-dose aspirin may affect tubular function, but overall renal clearance is low	Mainly metabolized in the liver, with low renal clearance	Mainly excreted *via* the kidneys	Renal clearance accounts for 25%–35%	Renal clearance accounts for approximately 80%
Nonrenal clearance	Low non‐renal clearance	Mainly metabolized in the liver	Mainly metabolized in the liver	High nonrenal clearance	Mainly metabolized in the liver	Mainly metabolized in the liver
Advantages	High bioavailability, long half-life, predictable effects, and low bleeding risk	Oral administration, low cost, and good long-term safety	Low cost and broad indications	Rapid onset and reversible	Oral administration and no routine monitoring is required	Oral administration and no routine monitoring is required
Disadvantages	Requires subcutaneous injection	Irritates the gastric mucosa and may cause gastrointestinal discomfort	Requires regular INR monitoring and interacts with many drugs and foods	Requires intravenous injection and has high bleeding risk	High cost	High cost
